# Association of C4d with disease activity in anti-neutrophil cytoplasmic antibody-associated vasculitis: evidence for classical/lectin complement pathway activation

**DOI:** 10.1186/s13075-025-03503-0

**Published:** 2025-03-05

**Authors:** Anna Juto, Myriam Martin, Albin Björk, Leonid Padyukov, Caroline Grönwall, Aleksandra Antovic, Annette Bruchfeld, Iva Gunnarsson, Anna M. Blom

**Affiliations:** 1 Present Address: Division of Rheumatology, Department of Medicine Solna, Karolinska Institutet and Karolinska University Hospital, Stockholm, Sweden; 2 Present Address: Department of Translational Medicine, Section of Medical Protein Chemistry, Lund University, Lund, Sweden; 3 Department of Clinical Chemistry and Pharmacology, Office for Medical Services, Region Skåne, Sweden; 4 Center for Rheumatology, Academic Specialist Center, Stockholm, Sweden; 5 Department of Health, Medicine and Caring Sciences, Linköping University, Linköping, Sweden; 6 Department of Renal Medicine, Karolinska University Hospitaland, CLINTEC Karolinska Institutet, Stockholm, Sweden

**Keywords:** Anti-neutrophil cytoplasmic antibody (ANCA)-associated vasculitis, Complement system, Classical pathway, Lectin pathway, C4d, ANCA serology

## Abstract

**Background:**

We aimed to investigate the involvement of the classical/lectin complement pathway in anti-neutrophil cytoplasmic antibody (ANCA)-associated vasculitis (AAV) by exploring the complement activation fragment C4d in association to AAV activity.

**Methods:**

Forty patients with active AAV and twenty population-based controls were included. The study included 27 (67.5%) patients with a diagnosis of GPA and 13 (32.5%) with MPA. Twenty-four patients (60%) were anti-proteinase 3 (PR3)-ANCA positive and 16 (40%) anti-myeloperoxidase (MPO)-ANCA positive. Thirty-three (82.5%) patients had kidney involvement. A follow-up sample obtained after induction therapy (median 6 months) was available for 24 of the patients, of whom 20 were in remission. Plasma C4d was analysed by ELISA detecting an epitope that arises upon complement-mediated cleavage. Plasma complement factor 4 (C4) and the soluble terminal complement complex (sTCC) were analysed by ELISA. The C4d/C4 ratio was calculated. HLA-DRB1-typing and immunohistochemistry for C4d in kidney biopsies were performed.

**Results:**

Patients with active AAV had higher C4d, sTCC levels and C4d/C4 ratio than controls (p < 0.001, p = 0.004, p < 0.001). C4d, sTCC levels and C4d/C4 ratio all decreased from active disease to remission (p = 0.010, p = 0.009, p = 0.011). C4d levels in AAV patients in remission remained higher than population-based controls (p = 0.026). Active anti-PR3-ANCA patients had higher C4d levels and C4d/C4 ratio than anti-MPO-ANCA patients (p = 0.001, p = 0.007). Patients with active AAV and kidney involvement had lower C4d levels than patients without (p = 0.04). C4d levels and C4d/C4 ratio correlated positively with the percentage of normal glomeruli in kidney biopsies. The immunohistochemistry was negative for C4d in kidney biopsies.

**Conclusions:**

The specific C4d assay revealed activity in the classical/lectin complement pathway in AAV, which reflected general disease activity, but was not associated specifically with kidney involvement. C4d levels differed depending on anti-PR3/MPO-ANCA subtypes suggesting differences in complement activation and underlying pathogenetic mechanisms. The findings imply that the classical/lectin complement pathway may play a more significant role in AAV pathogenesis than previously reported and that plasma C4d levels and C4d/C4 ratio may be biomarker candidates for disease activity and treatment outcome monitoring.

**Supplementary Information:**

The online version contains supplementary material available at 10.1186/s13075-025-03503-0.

## Background

Granulomatosis with polyangiitis (GPA) and microscopic polyangiitis (MPA) belong to the group of autoimmune necrotizing small vessel vasculitides, anti-neutrophil cytoplasmic antibody (ANCA)-associated vasculitis (AAV) [1]. GPA and MPA differ to some extent in genetics, clinical presentation and ANCA serology but are commonly regarded as a single disease entity with similar treatment and follow-up approaches [2–4]. Approximately 65–75% of GPA patients are anti-proteinase 3 (PR3)-ANCA positive and 20–30% have anti-myeloperoxidase (MPO)-ANCA while 20–30% of patients with MPA express anti-PR3 (αPR3)-ANCA and 55–65% are anti-MPO (αMPO)-ANCA positive [1]. AAV pathogenesis is complex, but activation of the alternative complement pathway has been shown to have a pivotal role in the pathogenic process [5, 6]. Activation of the alternative pathway has been confirmed in studies of blood, urine, and kidney tissue of AAV patients as well as in murine models of the disease [6, 7]. However, evidence supporting the contribution of the classical or lectin complement pathways is limited [8, 9].

Complement factor 4 (C4) and its final activation fragment C4d are components of the classical and lectin pathways. The terminal complement complex is the product of the three converging complement pathways [5], which occur on cell surfaces forming a lytic pore but can also be detected in blood in a soluble form called soluble terminal complement complex (sTCC, sC5b-9)[10]. C4d has shown promise as a complement activation biomarker in many contexts due to its ability to bind covalently to free hydrogen groups at sites of complement activation [11].

C4d has previously been assessed in AAV patients where plasma C4d levels were higher in AAV patients with active disease and in remission compared to healthy controls [12]. Still, no significant difference was seen between patients with active disease and remission [9, 12].

Previous studies have also shown C4d deposits in AAV kidney biopsies, suggesting that the classical and/or lectin pathways are part of the pathogenic process in AAV with kidney involvement. The depositions were primarily identified in glomeruli [13–16]. Additionally, C4d depositions were detected in interlobular arteries, peritubular capillaries and venules [14, 15].

The genes encoding for C4, *C4A* and *C4B*, are located on chromosome 6 within the MHC/complement cluster [17, 18]. The ancestral haplotype, AH8.1, carries HLA-DRB1*03:01 allele, lacks the *C4A* gene, contains a short *C4B* gene and includes HLA-B*08:01 allele. This haplotype is associated with the C4 levels in plasma [19, 20]. In the European population, variation in the C4 copy number, along with several SNPs, affects gene expression, thereby influencing C4 protein isotypes representation in plasma [17, 18].

sTCC was reported to be increased in AAV patients with active disease compared to patients in remission and controls [12]. However, conflicting data was conveyed in another study with no difference observed using the same antibody supplier [9]. Moiseev et al. used another antibody for sTCC detection and found similar results with no significant difference between active disease and remission [21].

Our objective was to examine the involvement of the classical/lectin complement pathway in AAV. We used a specific ELISA to detect a neoepitope of C4d that is exclusively formed by complement-mediated cleavage and investigated the complement cleavage fragment C4d as a potential biomarker of AAV disease activity. C4 and sTCC were also analysed for comparison of activity within the complement system.

## Methods

### Patients and sample collection

Forty patients in active disease phase from the vasculitis cohort at Karolinska University Hospital, between 2009–2019, with a clinical diagnosis of either GPA or MPA were included in the study. At inclusion, EDTA plasma was collected and cryopreserved within four hours of sampling and stored at -70 $^\circ{\rm C}$. All samples had been thawed at batching and separate aliquots for each planned analysis were sent to the collaborators. Twenty-four of the patients had follow-up samples available after induction treatment, with a median (interquartile range, IQR) of 6,0 (5,0-7,75) months between samplings. For comparison, 20 population-based age-matched controls were included from a large biobank of population-based controls. The controls had a median (IQR) age of 55 (49–60) years and seven (35%) were men.

### Disease activity and definition of kidney involvement

Clinical data was obtained from medical records. BVAS version 3 [22] was used to assess vasculitis disease activity. Disease remission was defined as a BVAS of 0 and active disease BVAS > 0. Kidney involvement was defined as pathological changes consistent with pauci-immune vasculitis in a recent kidney biopsy and/or significant haematuria (defined as ≥ 2 on dipstick urinalysis or ≥ 10 erythrocytes per high-power field on urinary sediment) and/or as a clinical presentation with decreased kidney function defined as a rise in serum creatinine > 30% or a fall in creatinine clearance > 25%.

Kidney biopsies were obtained at disease presentation in all 33 patients with signs of kidney involvement as defined above. The biopsies were assessed and categorized according to histopathologic classification [23] at the Department of Pathology at the Karolinska University Hospital. Moreover, the percentage of normal glomeruli [24] and the percentage of glomeruli with cellular and fibro-cellular crescents or necrosis or segmental sclerosis, as well as the percentage of sclerotic glomeruli of total glomeruli were assessed in the kidney biopsies.

### C4d assay

Plasma C4d was analysed using ELISA (COMPL C4d RUO, SVAR Life Science, Sweden), which targets a specific neoepitope in C4d (including both A and B genetic variants) that appears solely upon complement-mediated cleavage. The local reference interval for healthy individuals was 0.038–0.196 mg/L.

### C4 and sTCC assays

Determination of plasma C4 levels was performed using an Optilite turbidimeter (The Binding Site) at the Department of Clinical Immunology, Karolinska University Hospital, with a normal concentration range between 0.13–0.32 g/L.

Plasma levels of sTCC were determined by ELISA (#COMPL TCC RUO; Svar Life Science) according to the manufacturer’s instruction with a local reference interval for a population of healthy individuals of 0.031–0.131 mg/L.

### Routine lab and serology

Plasma creatinine, C-reactive protein (CRP), urine dipstick, urine sediment, and urine-albumin creatinine ratio (uACR) were analysed at the Departments of Clinical Chemistry and Immunology, Karolinska University Hospital as part of clinical routine, also including ANCA (αPR3, αMPO) autoantibody testing. The estimated glomerular filtration rate (eGFR) was calculated using the revised Lund-Malmo GFR (glomerular filtration rate) estimating equation (LM-rev) [25].

### Immunohistochemistry in kidney biopsies

Kidney biopsies of ten patients were available for immunohistochemistry (IHC) of C4d depositions. IHC was performed with the same anti-C4d-neo monoclonal Ab (dilution 1:5000, SVAR Life Science) used in the C4d assay in plasma. The staining procedure followed the previously described protocol [26]. A patient with lupus nephritis (LN) was used as a staining control.

### HLA-DRB1 typing

HLA-DRB1 genotyping was performed using the DR Low Screening kit (Olerup SSP, CareDX AB, Sweden). After PCR and electrophoretic separation of the amplicons on an agarose gel with GelRed, DRB1 genotypes were assigned according to the manufacturer's interpretation table.

### Statistical analysis

Descriptive statistics were used for patients’ characteristics; median (interquartile range, IQR) or median (min–max) for continuous data. Histograms and Shapiro-Wilks test were used to examine distributions. The results of complement levels are reported as median (IQR). Mann–Whitney U test was used for group comparisons when the data comprised ordinal data or were not normally distributed. Fisher’s exact test was used for categorical variables. Spearman’s rho was computed for correlation analysis. For comparison between > 2 groups Kruskal–Wallis test was performed with correction for multiple comparisons using Dunn-Bonferroni approach. For comparison between two related groups Wilcoxon signed ranks test was utilized. C4d/C4 ratio was calculated with both variables in mg/L. Statistical significance was defined as *p* < 0.05. Statistical analyses were performed with SPSS (IBM Corp. Released 2021. IBM SPSS Statistics for Windows, Version 28.0. Armonk, NY: IBM Corp).

## Results

Of the 40 active patients included, 39 were newly diagnosed and one had a relapsing disease. Eighteen patients were males (45%), 24 (60%) were αPR3-ANCA positive and 16 (40%) were αMPO-ANCA positive. The study included 27 (67.5%) patients with a diagnosis of GPA and 13 (32.5%) with MPA. The patients with GPA and αPR3-ANCA positivity were the same, except for three who had αMPO-ANCA. Thirty-three (82.5%) patients had kidney involvement as defined above, all biopsy confirmed. There was no difference in the frequency of kidney involvement between patients based on diagnosis or ANCA subtype. The patients' demographics and detailed characteristics are listed in Table 1. Patients with active disease and GPA or αPR3-ANCA were significantly younger (p = 0.018, p = 0.011) and had better eGFR (p = 0.004, p = 0.004) compared to patients with MPA or αMPO-ANCA positivity (Table 1).

**Table 1:** Patients’ characteristics

	**Baseline, all active patients **		**Subgroup with paired analysis**		
			**Baseline**	**Post-induction therapy**	**p**
	**n (%) and/or median (IQR)**				
N	40^a^		20^a^^,b^	20	
Sex, male	18 (45)		7 (35)	-	
**Age,** years	55 (49–64)		60 (42–65)	-	
GPA	53 (42–58)	p = 0.018			
MPA	63 (56–67)				
αPR3-ANCA positivity	53 (41–58)	p = 0.011			
αMPO-ANCA positivity	63 (54–68)				
**Disease characteristics**					
GPA	27 (67.5)		11 (55)	-	
MPA	13 (32.5)		9 (45)	-	
αPR3-ANCA positivity	24 (60)		11 (55)	5 (25), Negative: 5 (25)	
αMPO-ANCA positivity	16 (40)		9 (45)	5 (25), Negative:3 (15)^d^	
BVAS	16 (13–21)		15 (11–19)	0^e^	
**eGFR**, *Malmö-Lund Revised,* *mL/min/1.73m*^*2*^	71.4 (31.1–86.1)		64.3 (22.9–87.1)	61.8 (29.3–80.1)	ns
GPA	81.1 (58.2–87.2)	p = 0.004			
MPA	28.8 (18.5–66.0)				
αPR3-ANCA positivity	81.8 (63.1–89.0)	p = 0.004			
αMPO-ANCA positivity	39.5 (21.2–69.2)				
Haematuria^c^	33 (82.5)		14 (70)	2 (10)	< 0.001
uACR, *mg/mmol*	7.1 (0.8–41.9), 23 (57.5)		7.7 (0.5–60.0) 14 (20)	18.6 (0.4–39.3) 16 (80.0)	ns
CRP, *mg/L*	29.0 (8–55)		17 (6–36)	1.0 (1–5)	0.002
**Organ involvement**					
General	38 (95)		19 (95)	0	
Kidney	33 (82.5)		14 (70)	0	
Ear-nose-throat	21 (52.5)		10 (50)	0	
Chest	19 (47.5)		10 (50)	0	
Mucous membranes/eyes	11 (27.5)		6 (30)	0	
Cutaneous	8 (20)		4 (20)	0	
Nervous system	5 (12.5)		1 (5)	0	
Abdominal	1 (2.5)		0	0	
Cardiovascular	0		0	0	
**Treatment upon sampling**					
None	11 (27.5)		7 (35)	0	
PO Prednisolone	23 (57.5)		8 (40)	20 (100)	
IV Methylprednisolone	16 (40)		9 (45)	0	
Cyclophosphamide	5 (12.5)		1 (5)	3 (15)	
Rituximab	0		0	1 (5)	
Methotrexate	2 (5)		1 (5)	6 (30)	
Azathioprine	1 (2,5)		1 (5)	4 (20)	
Mycophenolate mofetil	0		0	3 (15)	

*eGFR* estimated glomerular filtration rate, *CRP* C-reactive protein, *GPA* Granulomatosis with polyangiitis, *MPA* microscopic polyangiitis, *αMPO* anti-myeloperoxidase, *ANCA* Anti-neutrophil cytoplasmic antibodies, *αPR3* anti-proteinase 3; uACR, Urine-Albumin/Creatinine ratio; BVAS, Birmingham Vasculitis Activity Score; PO, Per os; IV, intravenous

^a^One patient had undetectable C4d levels and was excluded from statistical analyses regarding C4d levels and C4d/C4 ratio

^b^Four patients were excluded since two patients had BVAS of 16 (Ear-nose-throat, Kidney and Ear-nose-throat, Chest, Nervous system), one patient had BVAS 2 (Mucous membranes) and one patient had BVAS 1 (Nervous system) post induction therapy

^c^Defined as moderate on urinalysis (≥ 2 +) or ≥ 10 RBC per high power field

^d^Missing data on ANCA status for two patients post-induction treatment

^e^VDI, vasculitis damage index, median (min–max) score 1 (0–3)

### Plasma C4d, sTCC levels and C4d/C4 ratio are increased in AAV patients

**Fig. 1:**
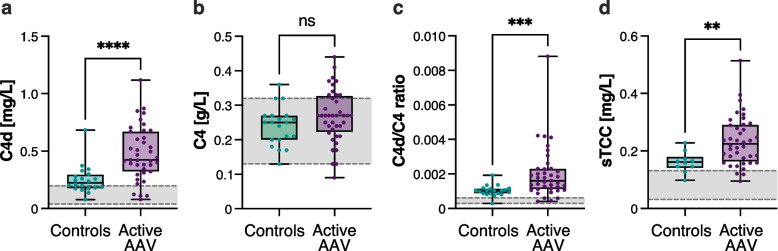
Plasma C4d, C4d/C4 ratio and sTCC levels are increased in patients with active ANCA-associated vasculitis (AAV). C4d (**a**), C4 (**b**), C4d/C4 ratio (**c**) and sTCC (**d**) levels in population-based control subjects (n = 20) and AAV patients with active disease (n = 40). The grey ranges depict the normal reference intervals. Data are presented as medians with 25–75% quantiles plus min to max whiskers, and significance was calculated using the Mann–Whitney U test. **p < 0.01, ***p < 0.001, ****p < 0.0001

At baseline, AAV patients displayed significantly higher levels of plasma C4d [median (IQR) 0.42 (0.32–0.67) mg/L vs 0.22 (0.17–0.30), p < 0.001, Fig. 1a] and C4d/C4 ratio compared to controls [0.0016 (0.0011–0.0023) vs 0.0010 (0.0009–0.0011), p < 0.001, Fig. 1c].

Moreover, sTCC levels were higher among patients with active disease compared to controls [0.23 (0.16–0.29) mg/L vs 0.16 (0.14–0.18), p = 0.004, Fig. 1d]. There was no significant difference in C4 concentrations between active patients and controls (Fig. 1b).

Post-induction therapy patients in remission (n = 19) had higher plasma C4d levels compared to controls [0.34 (0.20–0.44) mg/L vs 0.22 (0.17–0.30), p = 0.026]. There were no differences in levels of C4, sTCC or C4d/C4 ratio between patients in remission and controls (data not shown). Patients with active disease showed a significant correlation between CRP and levels of C4d (r_s_ = 0.46, p = 0.003), sTCC (r_s_ = 0.37, p = 0.02) as well as C4d/C4 ratio (r_s_ = 0.36, p = 0.025). No significant correlation was seen between CRP and C4 levels. One patient had undetectable C4d levels and was excluded from the statistical analysis regarding C4d levels and C4d/C4 ratio.

### Plasma C4d level and C4d/C4 ratio are higher in αPR3- than in αMPO-ANCA positive patients

**Fig. 2:**
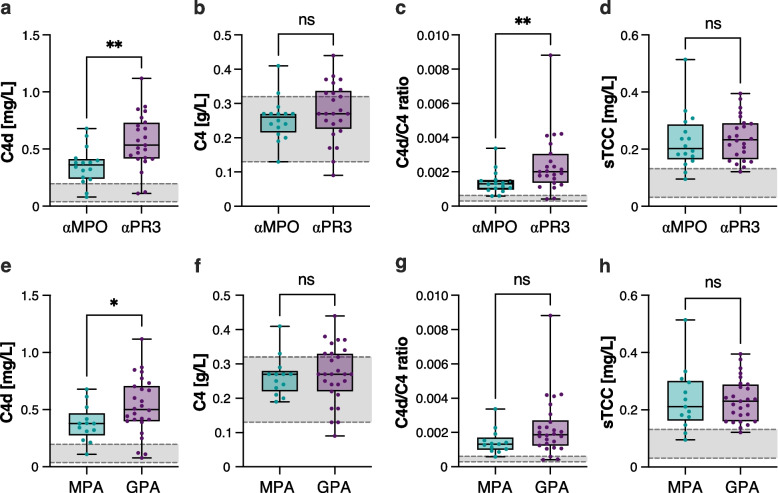
Plasma C4d levels are increased in active ANCA-associated vasculitis (AAV) patients with a diagnosis of granulomatosis with polyangiitis (GPA) or anti-proteinase 3 (PR3) ANCA positivity. C4d (**a**), C4 (**b**), C4d/C4 ratio (**c**) and sTCC (**d**) levels in anti-myeloperoxidase (αMPO, n = 16) positive versus anti-PR3 (αPR3) autoantibody positive (n = 24) AAV patients with active disease. C4d (**e**), C4 (**f**), C4d/C4 ratio (**g**) and sTCC (**h**) levels in GPA patients (n = 27) versus microscopic polyangiitis (MPA, n = 13) patients with active disease. The grey ranges depict the normal reference intervals. Data are presented as medians with 25–75% quantiles plus min to max whiskers, and significance was calculated using the Mann–Whitney U test. **p < 0.01

αPR3-ANCA positive patients had significantly higher C4d levels [median (IQR) 0.53 (0.41–0.73) mg/L vs 0.36 (0.24–0.41), p = 0.001, Fig. 2a] and C4d/C4 ratio compared to patients with αMPO-ANCA [0.0020 (0.0014–0.0031) vs 0.0013 (0.001–0.002), p = 0.007, Fig. 2c]. Likewise, GPA patients displayed significantly higher C4d levels compared to MPA patients [0.50 (0.40–0.71) mg/L vs 0.38 (0.27–0.47), p = 0.029, Fig. 2e], but there was no difference in C4d/C4 ratio (Fig. 2g). Based on diagnosis or ANCA autoantibody subset, there was no significant difference in levels of C4 (Fig. 2b, f) or sTCC (Fig. 2d, h).

Levels of αPR3-ANCA (n = 18) correlated positively with C4d levels (r_s_ = 0.49, p = 0.038) and C4d/C4 ratio (r_s_ = 0.65, p = 0.003), which was not observed for αMPO-ANCA titers (n = 12), C4 or sTCC levels (n = 12).

### ANCA serology and kidney involvement influence C4d levels

**Fig. 3:**
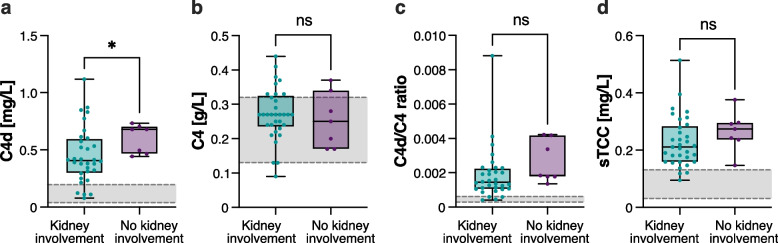
Plasma C4d levels are lower in active ANCA-associated vasculitis (AAV) patients with kidney involvement. C4d (**a**), C4 (**b**), C4d/C4 ratio (**c**) and sTCC (**d**) levels in active AAV patients with kidney involvement (n = 33) versus active AAV patients without kidney involvement (n = 7). The grey ranges depict the normal reference intervals. Data are presented as medians with 25–75% quantiles plus min to max whiskers, and significance was calculated using the Mann–Whitney U test. *p < 0.05

Patients with kidney involvement had significantly lower C4d levels compared to those without kidney involvement [median (IQR) 0.40 (0.30–0.60) mg/L vs 0.68 (0.47–0.70), p = 0.04, Fig. 3a]. There was no significant difference in levels of C4 (Fig. 3b), sTCC (Fig. 3d) or C4d/C4 ratio (Fig. 3c) between patients with and without kidney involvement. As expected, patients with kidney involvement had significantly higher BVAS than patients without [17 (15–22) vs 11 (7–11), p < 0.001].

Patients with kidney involvement were further subdivided into two groups based on C4d levels, with the cut-off based on the median level, ≥ 0.407 mg/L (low group, n = 16; high group, n = 16). In the high C4d level group, 13 patients (81.3%) were αPR3-ANCA positive and three αMPO-ANCA positive, whereas in the low C4d level group four patients (25%) had αPR3-ANCA and 12 patients (75%) αMPO-ANCA (p = 0.004). There was no difference in sex, diagnosis, or ongoing treatment between the low and high C4d groups. Moreover, there were no statistically significant differences in eGFR, BVAS, or uACR between the groups (data not shown).

There was no significant correlation between BVAS and C4d, C4, sTCC levels or C4d/C4 ratio (data not shown).

### Plasma C4d level and C4d/C4 ratio associate with disease activity

For paired analysis when including all patients (n = 23) there was a statistically significant reduction in levels of C4d [median (IQR) 0.47 (0.38–0.68) mg/L vs 0.30 (0.20–0.43), p = 0.003] and C4d/C4 ratio [0.0018 (0.0013–0.0021) vs 0.0011 (0.0008–0.0017), p = 0.002] post-induction treatment, but there was no significant difference in C4 levels. Post-induction treatment, 20 of the 23 patients were in remission.

**Fig.4:**
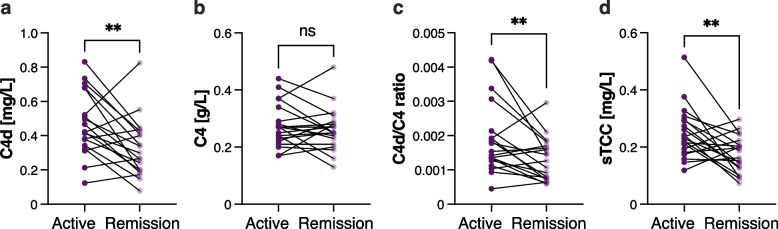
Plasma C4d, C4d/C4 ratio and sTCC levels are lower in remission in ANCA-associated vasculitis (AAV) patients than in active patients. C4d (**a**), C4 (**b**), C4d/C4 ratio (**c**) and sTCC (**d**) levels in active AAV patients at baseline in comparison to patients in remission after post-induction therapy. Data are presented as paired individual samples, and significance was calculated using Wilcoxon matched-pairs signed rank test. **p < 0.01

There was a significant reduction in C4d levels [median (IQR) 0.42 (0.34–0.68) mg/L vs 0.30 (0.20–0.43), p = 0.010, Fig. 4a] and C4d/C4 ratio [0.0015 (0.0013–0.0021) vs 0.0013 (0.0007–0.0017), p = 0.011, Fig. 4c] between patients in active disease state and after induction therapy when only including those in remission (n = 19), thus four patients who did not achieve remission were excluded from the analysis. However, no significant difference was seen in C4 levels between the two disease states (n = 20) (Fig. 4b).

Six patients had increased C4d levels, and an increased C4d/C4 ratio post-induction treatment compared to baseline. Among these patients, one experienced Herpes-Zoster infection two days after blood sampling. There was no difference in eGFR, CRP, or BVAS at baseline sampling, diagnosis, ANCA autoantibody subset, or improvement in albuminuria between patients based on increased or decreased C4d levels or C4d/C4 ratio in remission or compared to when in active disease state. The six patients with higher C4d levels and C4d/C4 ratio were still in remission six months after the six-month samples were obtained, following induction treatment.

### Plasma sTCC level associate with disease activity

sTCC levels were significantly decreased post-induction treatment when including all patients (n = 24) [median (IQR) 0.23 (0.18–0.30) mg/L vs 0.18 (0.13–0.22), *p* = 0.006], likewise when only including patients in remission (n = 20) [0.23 (0.18–0.30) mg/L vs 0.18 (0.13–0.22), p = 0.009, Fig. 4d]. Six of the 20 patients in remission at follow-up sampling, post-induction treatment, exhibited elevated plasma sTCC levels compared to active disease and remained in remission during the following six months. We observed no differences with regard to eGFR, change in eGFR, albuminuria, CRP, diagnosis or BVAS (in active disease) between patients with higher levels compared to lower sTCC levels post-induction treatment (data not shown).

### Type of immunosuppressive therapy does not affect complement levels

Eleven patients did not have ongoing immunosuppressive therapy at baseline (Table 1). Their C4d, C4, sTCC levels and C4d/C4 ratio were not statistically different from those on treatment (Additional file 1, Table 1). There was no significant difference in levels of the complement markers between patients based on the different immunosuppressive therapies (Additional file 1, Table 2).

### C4d level and C4d/C4 ratio correlate with kidney function and urine variables

There was a significant positive correlation between eGFR and C4d levels (r_s_ = 0.47, p = 0.003) as well as C4d/C4 ratio (r_s_ = 0.54, p < 0.001) among patients with active disease. Similar results on positive correlations with eGFR were seen when analysing patients with kidney involvement, C4d levels (r_s_ = 0.35, p = 0.048) and C4d/C4 ratio (r_s_ = 0.50, p = 0.003). No significant correlation was seen between eGFR and C4 or sTCC levels for any of the patient groups. uACR correlated negatively with C4d (r_s_ = -0.45, p = 0.034). No correlation was seen between uACR and C4, sTCC levels or C4d/C4 ratio.

### C4d level and C4d/C4 ratio correlate with kidney biopsy findings

The activity of kidney involvement was evaluated by assessing the percentage of normal glomeruli, the percentage of glomeruli with cellular and fibro-cellular crescents or necrosis or segmental sclerosis, and the percentage of sclerotic glomeruli of total glomeruli in the kidney biopsies, in relation to C4d, C4, sTCC levels and C4d/C4 ratio. Both C4d levels and C4d/C4 ratio correlated positively with the percentage of normal glomeruli and negatively with the percentage of necrosis or crescents in the biopsies (Table 2). There were no significant associations between C4 or sTCC levels and the percentage of affected glomeruli.

**Table 2:** Correlations between complement levels and kidney biopsy findings

*Correlations*	Percentage normal glomeruli of total glomeruli	Percentage glomeruli with cellular and fibro-cellular crescents or necrosis or segmental sclerosis of total glomeruli
C4d, n = 32	**r**_**s**_** = 0.380, *p* = 0.032**	**r**_**s**_ **= -0.463,** ***p*** **= 0.008**
C4, n = 33	r_s_ = 0.07, p = 0.70	r_s_ = -0.14, p = 0.44
C4d/C4 ratio, n = 32	**r**_**s**_** = 0.438, *p* = 0.012**	**r**_**s**_** = -0.463, *p* = 0.008**
sTCC, n = 33	r_s_ = 0.093, p = 0.61	r_s_ = 0.185, p = 0.302

Kidney biopsies from 30 patients were classified according to histological type according to the Berden classification [23] as crescentic (n = 5), focal (n = 19), mixed (n = 3) and sclerotic (n = 3). There were no significant differences in levels of C4d, C4, sTCC, or C4d/C4 ratio between these groups (Table 3).

**Table 3:** Kidney biopsy classification in relation to complement levels

Median (min–max)	**Focal,** n = 19^a^	**Crescentic,** n = 5	**Mixed,** n = 3	**Sclerotic,** n = 3	**p**
C4d, *mg/L*	0.42 (0.08–1.12)	0.37 (0.11–0.77)	0.38 (0.34–0.40)	0.42 (0.21–0.61)	ns
C4, *g/L*	0.27 (0.13–0.44)	0.27 (0.09–0.41)	0.27 (0.27–0.33)	0.27 (0.21–0.29)	ns
C4d/C4 Ratio	0.0019 (0.0006–0.004)	0.0009 (0.0004–0.009)	0.0012 (0.001–0.0015)	0.0014 (0.001–0.0022)	ns
sTCC, *mg/L*	0.21 (0.121–0.35)	0.23 (0.14–0.51)	0.18 (0.095–0.09)	0.15 (0.12–0.18)	ns

^a^n = 18 for C4d and C4d/C4 ratio analyses

When patients with kidney involvement were subdivided into two groups based on C4d levels, as previously defined, the patients in the high C4d level group had a lower percentage of sclerosed glomeruli of total glomeruli [median (IQR) 7.3 (5.3–16.1) % vs 21.8 (13.8–32.1), p = 0.023] and a lower percentage of glomeruli with cellular and fibro-cellular crescents or necrosis or segmental sclerosis of total glomeruli than the C4d low group [14.3 (9.63–26.3) vs 28.2 (20.2–44.1), p = 0.019].

**Fig. 5:**
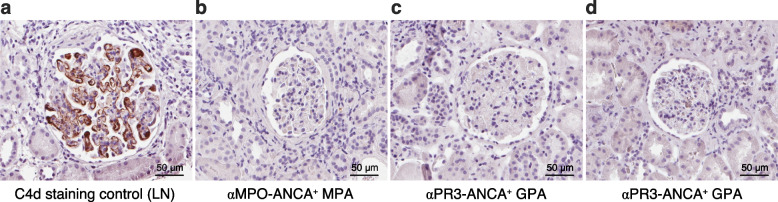
Immunohistochemistry shows absence of C4d depositions in kidney biopsies. Immunohistochemistry staining of C4d deposition in kidney biopsies using the same anti-neoC4d monoclonal Ab as applied in the C4d assay. A patient with lupus nephritis (LN) was used as a staining control (**a**). Representative images of three different kidney biopsies from two AAV patients are shown (**b-d**). A patient with αMPO-ANCA positive MPA with crescentic glomerulonephritis (**b**) and another patient with αPR3-ANCA positive GPA and focal necrotizing glomerulonephritis (FGN) (**c**) who had a repeat biopsy obtained after approximately nine months showing signs of previous FGN and no evidence of active inflammation (**d**). The C4d staining was negative in all investigated AAV biopsies

Immunohistochemical analysis of C4d depositions was performed on kidney biopsies from seven patients with active AAV and kidney involvement (αPR3-ANCA positive GPA, n = 5; αMPO-ANCA positive MPA, n = 2) with median (IQR) eGFR of 30 (21–87) mL/min/1.73 m^2^. Four patients had not started immunosuppressive treatment, two received IV methylprednisolone, and one patient received both IV methylprednisolone and one dose of IV cyclophosphamide before the kidney biopsy. Crescentic glomerulonephritis (GN) was seen in five patients and focal necrotizing GN was seen in two patients. Three of them (GPA n = 1, MPA n = 2) had a second kidney biopsy after median (min–max) of 9 (7–11) months. The repeat biopsy was performed due to increasing proteinuria in one of the MPA patients, whereas in the other two patients it was done as a clinical routine after induction therapy before changing to maintenance therapy, all displayed no active GN. The staining was negative in all 10 investigated biopsies. Three representative images are shown from two patients. One of them had αMPO-ANCA positive MPA with crescentic GN (Fig. 5b) and the other patient had αPR3-ANCA positive GPA with focal necrotizing GN (Fig. 5c) and a repeat biopsy obtained after approximately nine months showing signs of previous GN and no evidence of active inflammation (Fig. 5d). The GPA patient had received IV methylprednisolone and one dose of IV cyclophosphamide, and the other patient was untreated prior to biopsy.

### Complement biomarkers do not associate with HLADRB1-variants

To better understand a potential connection between the C4d levels and ANCA subtype, HLA-DRB1 alleles were analysed in all patients and controls. Eight patients and one control were carriers of the HLA-DRB1*03 allele, all in a heterozygous state. We found no significant difference in the frequency of HLA-DRB1*03 carriers between patients based on diagnosis, ANCA subtypes, sex, or kidney involvement (data not shown). Among patients with active disease, there were no differences in levels of C4d, C4, sTCC or C4d/C4 ratio between HLA-DRB1*03 carriers and non-carriers. Among patients with kidney involvement, the frequency of HLA-DRB1*03 carriers did not differ significantly between histological types or between groups with low and high C4d levels.

## Discussion

Plasma C4d levels were significantly increased in patients with active AAV disease compared to controls, especially in αPR3-ANCA-positive patients with non-renal involvement. Furthermore, we found high C4d/C4 ratio and sTCC levels in AAV patients with active disease, thus reflecting activity of all three complement pathways. Moreover, paired analyses post-induction treatment revealed that patients in remission had significantly decreased C4d, C4d/C4 ratio and sTCC levels, which contrasts with previously published data [9, 12]. This discrepancy might partially be explained by the fact that we were using an improved and more specific C4d assay and a newer sTCC assay.

Subdividing patients by autoantibody profile, we found that αPR3-ANCA positive patients had both higher C4d levels and C4d/C4 ratio compared to αMPO-ANCA positive patients, while no difference was observed for sTCC or C4 levels. A study concluded that αPR3-ANCA-positive patients, regardless of disease activity state, had significantly higher plasma C4d levels than healthy controls, which was not seen in αMPO-ANCA-positive patients [9]. Thus, our findings confirm previous data on differences in the role of C4d in specific ANCA subtypes [9], but also add new knowledge regarding disease activity and phenotypes. Notably, there was no statistically significant difference in the prevalence of kidney involvement based on ANCA-autoantibody subset or diagnosis. Not unexpectedly, GPA and αPR3-ANCA-positive patients had better kidney function than MPA and αMPO-ANCA-positive patients, thus consistent with previous studies [27]. Further subdividing patients with kidney involvement into two groups based on high or low C4d levels, we found that the majority of the C4d high group consisted of αPR3-ANCA positive patients.

C4d depositions have been found in the central necroses and surrounding palisading CD68-positive macrophages in other diseases where granulomas are present such as rheumatic noduli and granuloma annulare [28]. Granulomatous inflammation is common in αPR3-ANCA-positive AAV but is almost non-existent in MPA or αMPO-ANCA-positive AAV [1, 29]. Given that the αPR3-ANCA-positive group had more pronounced C4 activation, reflected by C4d levels and C4d/C4 ratio, suggests differences in the role of complement activation between the two ANCA-subsets.

It was shown previously that wild-type and C4-deficient mice developed GN upon exposure to anti-MPO IgG, whereas C5- and factor B-deficient mice did not [30]. Thereafter it has been discussed if the classical and lectin pathways are of importance in the pathogenesis of AAV, and indeed C4d depositions have previously been found in AAV GN in different areas of the kidneys as previously stated. The classical and lectin pathways are furthermore involved in the removal of apoptotic and necrotic cells and may hence be generally found in areas of tissue damage.

It has been suggested that the simultaneous presence of C4d and complement component 1q (C1q) deposits in kidney biopsies indicates activation of the classical complement pathway. In contrast, the presence of C4d without C1q would be more indicative of activation of the lectin pathway, particularly in the presence of mannose-binding lectin (MBL) [8, 14]. Studies analysing C1q and MBL in kidney biopsies of AAV patients with renal involvement, alongside C4d deposits, have demonstrated inconsistent results, ranging from negative findings to a low frequency of positive cases [13, 14, 16].

After kidney transplantation, tissue staining of C4d is used as one of the biomarkers of antibody-mediated rejection of kidneys [31]. Plasma C4d levels have also been shown to correlate with disease activity in systemic lupus erythematosus and increase prior to a flare in lupus nephritis, while also correlating with glomerular C4d depositions in lupus nephritis using the same method for C4d analysis [26, 32]. In previous AAV studies investigating kidney biopsies, C4d deposits have been found in different kidney compartments [13–16]. Here, one must keep in mind that when using antibodies that were elevated against antigens exposed on the C4d domain of C4 are reactive to all C4d-containing fragments of C4 including the intact molecule and C4b. Since the antibody we used exclusively binds to a cleavage neoepitope in C4d, and not against C4 or C4b, it is expected to yield a different staining pattern. In one of the previous studies, mainly consisting of αMPO-ANCA positive patients with impaired kidney function, 34 of 48 kidney biopsies had detectable C4d in the glomeruli. Notably, no significant difference between histopathological classes or ANCA subtype was found [13]. In another study, all AAV patients with kidney involvement (MPA n = 17 and GPA n = 2) had C4d depositions in the glomeruli where the staining findings varied depending on the histology of the affected glomeruli. However, ANCA serotypes, treatment and eGFR of the included patients were not specified [14]. In the study by Hakroush et al. kidney staining using anti-C4d antibodies was positive in a majority of AAV patients. All patients had ongoing glucocorticoid therapy and had impaired kidney function [15]. Additionally, a majority of αMPO-ANCA positive MPA patients with markedly impaired kidney function (median eGFR 17 mL/min/1.73 m^2^) had C4d glomerular deposits [16]. In contrast to these findings, using biopsies from seven AAV patients with either focal necrotizing or crescentic GN, we did not detect any C4d deposits using the antibody that targets a specific epitope in C4d, as described previously. Of note, strong glomerular C4d staining in kidney biopsies from lupus nephritis was shown using the same method [26], supporting the fact that the antibody indeed can be utilized to detect C4d within the kidneys. Our findings thus point to the fact that activation of the classical/lectin complement pathways AAV may mainly occur in the circulation or potentially locally in non-renal compartments in αPR3-ANCA-positive patients. Although, the contrasting results on tissue deposition could be explained by the usage of different antibodies and staining methods, the effects of treatment, disease severity and duration should also be considered. Unfortunately, these data are largely lacking in previous studies [13–16] thus hampering the possibility to make direct comparisons.

Patients without kidney involvement had higher C4d levels compared to patients with kidney involvement. Moreover, a positive correlation between eGFR and C4d levels was observed together with a positive correlation with the proportion of normal glomeruli in kidney biopsies. These findings could additionally reflect an underlying pathogenetic difference based on the degree of kidney involvement and/or ANCA subtype. AAV and in particular AAV with glomerulonephritis has mainly been characterized by alternative pathway activation and drug development has built on that discovery [30, 33]. The finding of lower C4d levels in patients with kidney involvement in our study needs to be confirmed in larger studies as one limitation of our study is the inclusion of only seven patients without kidney involvement. However, if this observation will hold, it could prompt an interesting hypothesis. C4d is a stable product of C4b cleavage by factor I, with co-factors such as C4b-binding protein (C4BP) or membrane cofactor protein (MCP) [5] and as such marks classical/lectin pathway activation in a specific organ (biopsies), systemically (C4d ELISA) or could be released from the affected tissues into blood. As we did not detect C4d in kidney biopsies in the current study, it is possible that patients with kidney involvement do not engage the classical/lectin complement pathway in kidneys. Instead, C4d may be upregulated systemically as a consequence of the inflammatory process in AAV or generated in other engaged organs. This is contrary to lupus nephritis where there is strong deposition of C4d in diseased kidneys and deposition levels correlated with C4d levels in blood [26]. Whether lower circulating C4d levels reflect more established kidney damage in AAV, as implied by the positive correlation between eGFR and C4d levels together with a correlation with the proportion of normal glomeruli in kidney biopsies or have any prognostic significance for eGFR loss needs to be further investigated in a larger longitudinal AAV cohort.

There was no statistically significant correlation between BVAS and C4d or sTCC levels, consistent with previous reports [9, 12, 21], nor with C4d/C4 ratio. Kidney involvement generally contributes to high BVAS scores and since the majority of the cohort had kidney involvement, this may have contributed to the fact that we did not see any significant correlation between BVAS and the levels of the different complement products.

The number of patients included was limited and patients with kidney involvement were overrepresented in the study, which affects the ability to draw conclusions on effects in specific AAV subtypes and disease manifestations. Of note, all patients with kidney involvement had undergone a kidney biopsy, thus we could both confirm and grade the kidney involvement.

Most of the patients had ongoing treatment at baseline sampling, which is a limitation of the study. As the disease requires prompt initiation of immunosuppressive treatment, the inclusion of treatment-naïve patients is challenging in studies of AAV. However, we found a significant decrease in C4d, sTCC levels and C4d/C4 ratio in patients post-induction therapy, which supports that the markers are affected by treatment and might thus be valuable to monitor treatment response especially now when the complement inhibitor avacopan is accessible under certain conditions as adjunctive treatment [34]. The dynamics of alterations in a short-term perspective need further studies and could add knowledge to the field.

## Conclusions

The specific C4d assay revealed activity of the classical/lectin complement pathway in AAV, which reflected general disease activity, but was not specifically associated with kidney involvement. C4d levels were dependent on ANCA-autoantibody profiles suggesting differences in the role of complement activation in the underlying pathogenetic mechanisms. This implies that the classical/lectin complement pathway may play a more significant role in AAV pathogenesis than previously reported and that plasma C4d levels and C4d/C4 ratio could potentially be used as biomarkers for monitoring disease activity and response to treatment. Larger studies on AAV including specific organ involvement and outcome could add to the utility of C4d in various AAV phenotypes.

## Supplementary Information

Supplementary file 1.

## Data Availability

No datasets were generated or analysed during the current study.

## Abbreviations

GPA: Granulomatosis with polyangiitis
MPA: Microscopic polyangiitis
ANCA: Anti-neutrophil cytoplasmic antibody
AAV: Anti-neutrophil cytoplasmic antibody (ANCA)-associated vasculitis
SNPs: Single nucleotide polymorphisms
