# Supplementary material for: Association of C4d with disease activity in anti-neutrophil cytoplasmic antibody-associated vasculitis: evidence for classical/lectin complement pathway activation
Source: Arthritis Res Ther. 2025 Mar 5;27:49. doi: 10.1186/s13075-025-03503-0 (PMC11881377; doi:10.1186/s13075-025-03503-0)
Supplement: Supplementary file 1 [file 13075_2025_3503_MOESM1_ESM.docx]

Additional file 1

**Table 1.** C4d, C4, sTCC levels and C4d/C4 ratio based on ongoing immunosuppressive treatment

| Median (IQR) | No ongoing immunosuppressive treatment, n = 11 | Ongoing immunosuppressive treatment, n = 29 ^a^ | p |
| --- | --- | --- | --- |
| C4d, mg/L | 0.47 (0.38-0.68) | 0.42 (0.27-0.61) | ns |
| C4, g/L | 0.25 (0.20-0.36) | 0.27 (0.23-0.30) | ns |
| C4d/C4 Ratio | 0.0018 (0.0013-0.0026) | 0.0015 (0.0012-0.0021) | ns |
| sTCC, mg/L | 0.27 (0.21-0.31) | 0.21 (0.16-0.27) | ns |

^a^ n = 28 for C4d and C4d/C4 ratio analyses.

**Table 2.** C4d, C4, sTCC levels and C4d/C4 ratio based on different ongoing immunosuppressive treatments

| Median (min-max) | No ongoing immunosup-  pressive treatment,  n = 11 | Prednisolone^a^, n = 9^b^ | Methylpred-nisolone ^c^,  n = 12 | Any GC+ CYC,  n = 5 | Any GC+ DMARD  (not CYC),  n = 3 | p |
| --- | --- | --- | --- | --- | --- | --- |
| C4d, mg/L | 0.47  (0.30-0.85) | 0.45  (0.08-0.67) | 0.42  (0.12-1.12) | 0.41  (0.11-0.87) | 0.52  (0.44-0.72) | ns |
| C4, g/L | 0.25  (0.13-0.41) | 0.28  (0.13-0.44) | 0.26  (0.09-0.37) | 0.27  (0.19-0.33) | 0.25  (0.17-0.27) | ns |
| C4d/C4 Ratio | 0.001 (0.0009-0.0042) | 0.0015  (0.0006- 0.002) | 0.0014  (0.0005-0.009) | 0.0013 (0.0004-0.004) | 0.0019 (0.0018-0.004) | ns |
| sTCC, mg/L | 0.27  (0.12-0.51) | 0.18  (0.10-0.25) | 0.21  (0.12-0.40) | 0.29  (0.14-0.35) | 0.19  (0.29-0.15) | ns |

GC, glucocorticoids; CYC, cyclophosphamide; DMARD, Disease-modifying antirheumatic drugs

^a^ Prednisolone, mg/day, median (min-max), at sampling, 40 (10-60).

^b^ n = 8 for C4d and C4d/C4 Ratio analyses.

^c^ IV methylprednisolone, mg/day, median (min-max), at sampling, 544 (80-1000).
